# Evaluating Soluble EMMPRIN as a Marker of Disease Activity in Multiple Sclerosis: Studies of Serum and Cerebrospinal Fluid

**DOI:** 10.1371/journal.pone.0163802

**Published:** 2016-10-11

**Authors:** Deepak K. Kaushik, Heather Y. F. Yong, Jennifer N. Hahn, Claudia Silva, Steven Casha, R. John Hurlbert, Francois H. Jacques, Robert Lisak, Omar Khan, Carolina Ionete, Catherine Larochelle, Alex Prat, Amit Bar-Or, V. Wee Yong

**Affiliations:** 1 Hotchkiss Brain Institute, University of Calgary, Calgary, Alberta, Canada; 2 CSSSG-Hull Pavilion, Gatineau, Quebec, Canada; 3 Multiple Sclerosis Center, Wayne State University School of Medicine, Detroit, Michigan, United States of America; 4 Multiple Sclerosis Center, University of Massachusetts, Worcester, Massachusetts, United States of America; 5 University of Montreal, Montreal, Quebec, Canada; 6 Montreal Neurological Institute, McGill University, Montreal, Quebec, Canada; University of Portsmouth, UNITED KINGDOM

## Abstract

*E*xtracellular *m*atrix *m*etallo*pr*oteinase *in*ducer (EMMPRIN, CD147) is an inducer of matrix metalloproteinases and has roles in leukocyte activation and migration. We reported previously that in MS and its animal model, experimental autoimmune encephalomyelitis, cell surface-associated EMMPRIN was significantly elevated in leukocytes around inflammatory perivascular cuffs in the CNS. In this study we report that activated T-cells can secrete soluble form of EMMPRIN (sEMMPRIN) upon activation. As sEMMPRIN is also present in biological fluids, we determined whether sEMMPRIN is altered in the CSF and sera of MS subjects. Sera from individuals without neurological conditions served as controls, while CSFs collected from subjects undergoing discectomy, and without evidence of CNS pathology, were used as a comparator group. We found that serum levels of sEMMPRIN from clinically stable MS patients or other inflammatory conditions did not differ from control subjects. Paired serum and CSF samples demonstrated poor correlation of sEMMPRIN. Interestingly, sEMMPRIN levels were approximately 60% higher in CSFs compared to sera. sEMMPRIN CSF levels were significantly higher in secondary progressive compared to primary progressive subjects. Thus we conclude that measurement of sEMMPRIN in serum is not informative for disease activity in MS. The differential expression of sEMMPRIN in the CSF of primary and secondary progressive MS invites hypotheses of the still undefined roles of EMMPRIN in the CNS.

## Background

Multiple sclerosis (MS) involves the influx of leukocytes into the central nervous system (CNS) leading to demyelination and axonal degeneration. Relapsing-remitting MS (RRMS) is the most common form of MS characterized by relapses interspersed with periods of clinical stability. A large number of RRMS patients eventually transition to secondary progressive MS (SPMS) where disability accumulates without obvious clinical relapses. Another progressive form of MS, primary progressive MS (PPMS), is diagnosed in about 10–15% of patients and is characterized by steady loss of function from onset without obvious relapse activity.

Among the various indicators of disease activity in the MS brain [1], adhesion molecules including vascular cell adhesion molecule-1 (VCAM-1) and intercellular cell adhesion molecule-1 (ICAM-1) are crucial for the adhesion and migration of T-cells along the vasculature [2, 3]. The expression of integrins and immunologlobulin (Ig) superfamily members is also important for leukocyte migration across the vascular endothelium [4]. In this context, the *E*xtracellular *M*atrix *M*etalloproteinase *I*nducer (EMMPRIN, CD147), a type I transmembrane glycoprotein of the immunoglobulin (Ig) superfamily, seems crucial as it can interact with adhesion molecules including integrins [5, 6]. Therefore, increased EMMPRIN expression on T-cells might facilitate their migration across barriers through engagement of adhesion molecules and through the reported capacity of EMMPRIN to increase the expression of several matrix metalloproteinases (MMPs) [7]. Indeed, we found that EMMPRIN was elevated on activated peripheral leukocytes during the onset and progression of experimental autoimmune encephalomyelitis (EAE), an inflammatory model of MS [8]. EMMPRIN was also elevated around perivascular cuffs in post-capillary venules of EAE and MS specimens where it was expressed on T and B-lymphocytes, and on monocytes; reactive astrocytes in proximity to these cuffs were also positive for EMMPRIN [9]. The upregulation of EMMPRIN in EAE and MS has pathological significance since the treatment of EAE mice with function blocking anti-EMMPRIN antibodies reduced the number of perivascular cuffs and clinical severity [8, 10].

In addition to its expression on the cell membrane, EMMPRIN can be secreted by cells and released into the extracellular space. This form of soluble EMMPRIN (referred to as sEMMPRIN hereafter) appears as a smaller fragment by virtue of processing by transmembrane type 1 (MT1)-MMP [11] or as a full-length molecule via vesicular release [12] and may have relevance in disease pathology. Indeed, sEMMPRIN in the sera of human subjects was found to be elevated in amyotrophic lateral sclerosis (ALS) [13] and systemic sclerosis [14] patients compared to healthy control subjects. Thus far, literature on the presence of sEMMPRIN in the CSF is lacking. Also, whether sEMMPRIN levels change during MS pathology remains to be elucidated.

## Methods

### Isolation of peripheral blood mononuclear cells (PBMCs) and activation of T-lymphocytes

#### Isolation of PBMCs

Blood was drawn from healthy adult human volunteers and PBMCs were purified using Ficoll-Plaque^™^ PLUS (GE Healthcare) centrifugation. Briefly, heparinized blood was diluted 1:1 with phosphate-buffered saline (PBS) and carefully layered over Ficoll and centrifuged. The cell interface layer was collected, washed twice in PBS and re-suspended in serum-free AIM-V media (Invitrogen Life Technologies, Burlington, Ontario, Canada) [10].

#### Activation of T-cells

Wells of a 96-well round bottom plate were coated with 1 μg/ml anti-human CD3 (BD Pharmingen) for 3h at 37°C. For non-activated T-cell conditioned media, wells were left uncoated. Isolated PBMCs were plated at 200,000 cells/well in a final volume of 100 μl. One μg/ml of soluble anti-human CD28 (BD Pharmingen) was also added to the anti-CD3 coated wells upon plating the PBMCs. This stimulation condition resulted in an enrichment of activated T-cells above 90% (data not shown). Conditioned media from each condition were harvested for EMMPRIN ELISA after 48h and 72h of activation.

### Measurement of sEMMPRIN and IgG

sEMMPRIN was analyzed from 5 μl of sera or CSF and 20 μl of conditioned media from in vitro cultures of PBMCs using the solid phase sandwich human EMMPRIN ELISA kit (R&D systems, Minneapolis, MN) according to the manufacturer’s protocol. The amount of sEMMPRIN was read off a standard curve generated from known quantities of EMMPRIN provided by the manufacturer. The standard curve was generated using the 4-parametric logistic curve-fit and the R^2^ value of 0.99 or higher was consistently observed. We note that the manufacturer was unable to provide information on the specific epitope that the antibody to EMMPRIN binds to, other than that the ELISA antibody is generated against the full-length form of EMMPRIN.

For IgG measurement, a 2000-fold dilution of CSF samples was analyzed using total human IgG ELISA kit (Affymetrix, Santa Clara, CA). All serum and CSF samples were measured in duplicate wells.

To address the reliability of sEMMPRIN measurements, we subjected aliquots of CSFs from 5 subjects to ELISA measurements conducted at 2 time periods spaced 20 months apart. The results in S1 Fig show that the same aliquot analyzed 20 months apart, and with different lot number of ELISA kits, gave similar values of sEMMPRIN.

### Sera and CSF samples

Serum samples from subjects of the following health conditions were: healthy controls (HC), Crohn’s disease (CD), ulcerative colitis (UC), type 1 diabetes (T1D), and RRMS. The ages (years ± SD) of these groups were, respectively, 31 ± 12, 25 ± 8, 33 ± 11, 26 ± 8, and 37 ± 12. All subjects were deemed clinically stable at the time of their blood collection.

For the paired serum and CSF study, these were collected from the same individuals considered “active”, with demyelinating event within the past 3 months, or “inactive”, without apparent demyelinating event the previous 3 months. Subjects had the following diagnosis: Subject 1 (S1)—other neurological disease (normal pressure hydrocephalus); S2—clinically isolated syndrome (CIS), active; S3—CIS, inactive; S4—CIS, active; S5- RRMS, active; S6—RRMS, active; S7- RRMS, active; S8- RRMS, active; and S9- RRMS, inactive. Subject S6 was in the midst of an acute relapse when specimens were obtained.

CSFs were collected by traditional lumbar punctures from 64 patients with RRMS (25), SPMS (21) and PPMS (10) and ALS (8) and stored at -80°C until further use. The age of most but not all patients was recorded at the time of the collection, and the known average ages (years ± SD) were 39 ± 11 (RRMS), 48 ± 7 (SPMS), 48 ± 7 (PPMS) and 58 ± 16 (ALS). Control CSF samples were collected by lumbar puncture under general anesthesia prior to microdiscectomy surgery (discectomy group). These 7 subjects were symptomatic with degenerative disc disease refractory to non-surgical management for >12 weeks but lacked evidence of CNS pathology on history and physical examination by a neurosurgeon. Their mean ages were 56 years ±17. Unlike the paired serum and CSF that were available from some individuals with MS, we did not have access to paired serum and CSF from the ALS subjects; thus, only CSFs were analyzed for the ALS group.

All the samples were collected from adult participants who provided their written informed consent to participate in this study, which was approved by local institutional ethics committee {The Conjoint Health Research Ethics Board (CHREB) of the University of Calgary}.

### Statistical analyses

Differences in variables between groups were tested by one-way ANOVA with Tukey’s post hoc test or Student’s t-test using Graphpad prism software. Correlation analysis was performed by Pearson correlation. Data is represented as mean **±** SD and sEMMPRIN as well as IgG values are expressed in pg/ml. p-values <0.05 were considered statistically significant. The minimal/main data set underlying the findings in our study is presented as S1 Table

## Results

### Activated T-cells release more sEMMPRIN than non-activated cells

**Fig 1:**
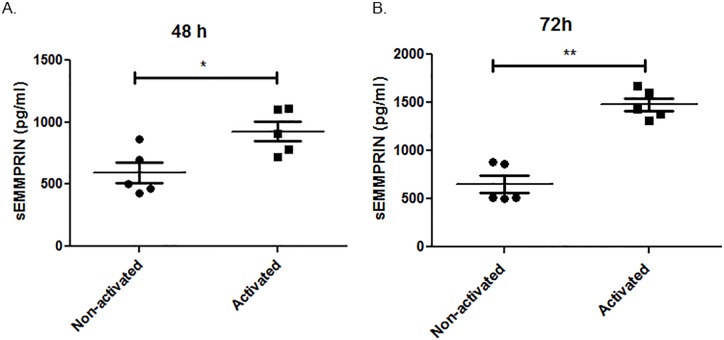
Increased sEMMPRIN secretion upon T-cell activation. PBMCs were isolated from five healthy subjects on three separate occasions and the T-cells were activated with anti-CD3 and anti-CD28 antibodies for 48 or 72h, or were left non-activated for the same time period. Conditioned media analyses found that activation of T-cells resulted in a progressive increase of sEMMPRIN level. The dots represent results from individual subjects, performed in duplicate. Comparison between the groups were done by Student’s t-test, *p<0.05 and **p<0.01. Values are represented in mean pg/ml ± SD.

We addressed whether sEMMPRIN levels alter upon activation of T-cells. Compared to non-activated conditions, we found that sEMMPRIN levels in conditioned media of T-cells activated with anti-CD3 and anti-CD28 were significantly elevated (Fig 1). These findings suggest that sEMMPRIN levels may be a useful indicator of the degree of cellular activation in inflammatory diseases.

### Serum sEMMPRIN does not differ amongst subjects with different inflammatory conditions

**Fig 2:**
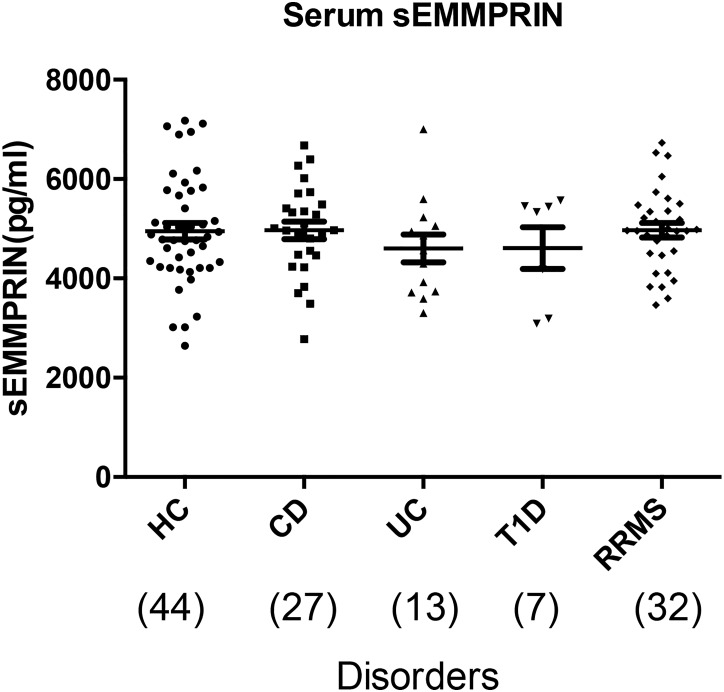
sEMMPRIN in sera was not altered in different inflammatory conditions. sEMMPRIN in sera from HC (healthy controls) and patients with CD (Crohn’s disease), UC (ulcerative colitis), T1D (Type 1 diabetes) or RRMS were measured by ELISA. The number of subjects is shown in parentheses. The scatter plot shows that sEMMPRIN levels did not significantly differ between the groups (one-way ANOVA with Tukey’s post hoc test). Values are represented in pg/ml, with each entry representing a single subject performed in duplicate, and data is represented as mean ± SD.

Earlier studies of ALS and systemic sclerosis had shown higher serum sEMMPRIN levels compared to healthy controls [13]. However, we found no evidence of alteration of serum sEMMPRIN levels in clinically stable RRMS subjects compared to healthy controls; levels in other inflammatory conditions including Crohn’s disease, ulcerative colitis and type 1 diabetes also were not different from healthy controls (Fig 2). The values of serum sEMMPRIN within a group were found to be highly variable; for example, the levels ranged between 2643–7175 pg/ml in healthy controls and between 3460–6730 pg/ml in RRMS subjects.

### CSF contains higher sEMMPRIN than serum

**Fig 3:**
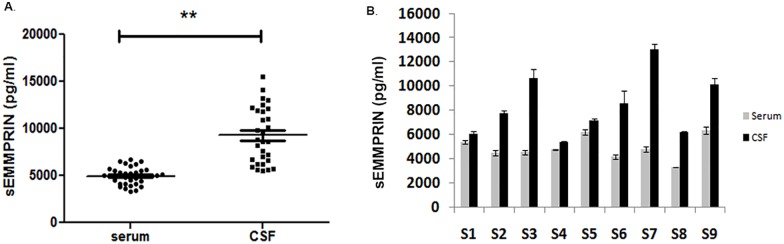
Comparisons of sEMMPRIN in CSF and serum samples of MS patients showed higher levels in the CSF. (A) Scatter plot of RRMS sera and CSFs from unpaired individuals show that sEMMPRIN levels were significantly higher in CSF compared to serum. Comparison between sera and CSFs done by Student’s t-test, **p<0.01. Individual values are represented in pg/ml and data is represented as mean ± SEM. (B) sEMMPRIN in paired sera and CSF from 9 subjects were measured by ELISA. S1 is a non-neurological control, while S2 to S9 were CIS or RRMS patients (see Methods). Subjects S2, 4, 5, 7 and 8 were considered active patients with a demyelinating event within the past 3 months, while Subjects S3 and 9 were inactive; Subject S6 was in the midst of an acute relapse. Sera and CSF sEMMPRIN levels were found to be poorly correlated in the matched specimens (Pearson correlation, r of 0.13, p = 0.74); error bars indicate differences from duplicate wells for each individual.

We analyzed sEMMPRIN levels in paired sera and CSF samples collected at the same draw from subjects along with unpaired sera and CSF samples from other MS collections. For the latter, we found that the CSF had statistically significant higher levels of sEMMPRIN over content found in serum samples (Fig 3A).

Further, in the majority of the paired samples collected from both the CIS and RRMS subjects, sEMMPRIN levels in the CSF was higher than in the corresponding serum samples (Fig 3B). However, the difference in the levels of sEMMPRIN between the paired sera and CSF samples was not uniform across subjects, and sEMMPRIN levels in the paired sera and the CSF between different subjects did not correlate well (r of 0.13; p = 0.74). Moreover, we could not find an obvious relationship between sEMMPRIN levels in active or inactive individuals.

### SPMS CSF has significantly higher sEMMPRIN levels over PPMS CSF

**Fig 4:**
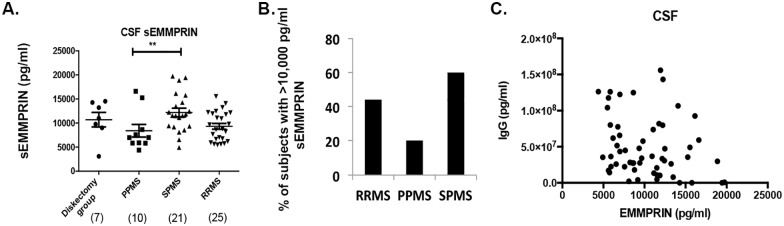
sEMMPRIN in CSF: higher levels in SPMS compared to PPMS subjects. (A) While CSF sEMMPRIN from the MS subtypes did not differ from discectomy group (3093.3–14530.0 pg/ml), the CSF sEMMPRIN from SPMS subjects (4928.4–19748.4 pg/ml) was statistically higher compared with PPMS CSF (4388.8–16614.4 pg/ml). Further, RRMS CSF sEMMPRIN levels (5617.9–15528.2 pg/ml) did not differ from other groups. Each entry represents the value from individual subjects performed in duplicate. Values are represented in pg/ml and the line data represents mean ± SEM. Groups were compared using one way ANOVA with Tukey’s post hoc test, **p<0.01. (B) Graph represents percentage of subjects in each group with sEMMPRIN levels >10,000 pg/ml. (C) Scatter plot showing correlation analysis between CSF IgG levels and sEMMPRIN in all the MS subjects. The sEMMPRIN in CSFs did not correlate with CSF IgG levels (Pearson correlation, r of -0.2382, p = 0.07).

When we measured sEMMPRIN in the CSF of individuals with different forms of MS, we observed large variability between the groups (Fig 4A). Notably, sEMMPRIN levels in the CSF of SPMS subjects were significantly higher over that of PPMS subjects (p < 0.05), although this may have resulted from a reduction in sEMMPRIN in the PPMS group as the result between SPMS and the discectomy group was not statistically significant. Amongst the groups, SPMS tended to have a higher percentage of subjects with greater than 10,000 pg/ml (we considered these values as ‘high sEMMPRIN’) for CSF sEMMPRIN (Fig 4B). However, when we compared CSF sEMMPRIN levels of MS subjects to the discectomy group, there was no statistical difference.

Furthermore, sEMMPRIN levels from all the MS subjects did not associate with their CSF IgG levels (Pearson correlation, r of -0.2382, p = 0.07). Also, sEMMPRIN levels did not differ significantly between male and female MS subjects (p = 0.07) as well as between different age groups (20–40 years versus 41–70 years, p = 0.08).

### CSF sEMMPRIN is not significantly altered in ALS subjects

**Fig 5:**
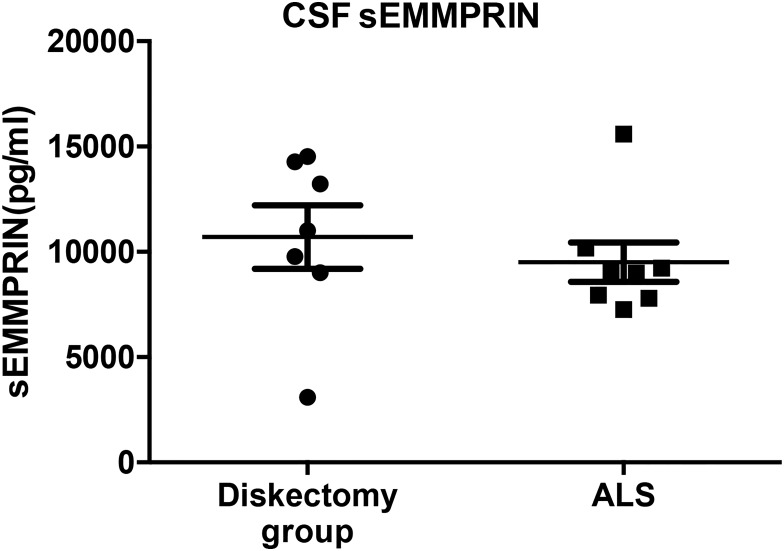
CSF sEMMPRIN levels in discectomy group and ALS patients are similar. Comparison of CSF sEMMPRIN in ALS and discectomy group revealed that there was no significant difference in sEMMPRIN levels between the two groups. Groups were compared by Student’s t-test; values are represented in pg/ml and data is represented as ±SD

CSF samples from 8 ALS patients were analyzed for differences in sEMMPRIN levels. In contrast to reports that sera sEMMPRIN is significantly higher in ALS subjects over healthy controls [13], we observed no such difference in sEMMPRIN levels in the CSF of ALS subjects over the discectomy group (Fig 5).

## Discussion

EMMPRIN is highly expressed in the brain capillary endothelium [15] and neuronal layers within the cortex and the cerebellum [16, 17]; genetic knockout studies have shown that EMMPRIN is crucial for proper functioning of the CNS [18, 19]. Through analyses of brain specimens, EMMPRIN has since been found to be associated with many diseased conditions of the CNS, including gliomas [20], Alzheimer’s disease [21], stroke/ischemia [22], and MS [8, 9]. A soluble form of EMMPRIN can be generated from the transmembrane full length protein, providing the possibility that sEMMPRIN may provide a measure of disease activity in living subjects, particularly since T-cells when activated in culture secrete more sEMMPRIN. sEMMPRIN is reported to be elevated in the sera of patients with ALS [13] and systemic sclerosis [14]; however, we did not find elevated sEMMPRIN in the CSFs of the ALS subjects. That EMMPRIN is not increased in the CSF of ALS subjects remains speculative but could be due to differences in cellular source of this protein in CSF compared to serum. Also, when examining serum sEMMPRIN levels in individuals with different inflammatory conditions compared to controls, no significant differences were found. Moreover, we found that serum sEMMPRIN did not correlate with relapse activity in MS. Thus, we conclude that the potential secretion of sEMMPRIN from immune cells into the circulation cannot be detected above the sEMMPRIN that may be derived from other unknown cellular and tissue sources.

We observed that serum sEMMPRIN levels were poorly correlated with CSF sEMMPRIN and that sEMMPRIN levels were markedly higher in the CSF compared to serum This suggests important roles of EMMPRIN in the blood-brain barrier interface or within the CNS. EMMPRIN can interact with different adhesion molecules on endothelial linings including integrins [5, 6], and it is an important modulator of lymphocyte migration across the CNS parenchyma [8, 9]. We speculate that ongoing inflammation can alter sEMMPRIN levels within the CSF, which is supported by our findings that activated T-cells are a potent source of sEMMPRIN. However, we did not find sEMMPRIN to be associated with relapse disease activity, although a more stringent definition of active disease should include MRI measurements and taking into account the possible effect of steroid treatment on disease activity and sEMMPRIN levels. We did not find sEMMPRIN and CSF IgG levels to have a significant correlation. Since previous work has shown that EMMPRIN expression is elevated on cells in active perivascular inflammatory cuffs in autopsied MS specimens, the lack of elevation of sEMMPRIN in the CSF during presumed active disease indicates that either membrane associated EMMPRIN in active MS brain lesions is not correlated with the cleaved sEMMPRIN form, or that sEMMPRIN drained into the CSF from active lesions is not detectable over the high levels already constitutive to the CSF compartment.

Our current findings show that sEMMPRIN is elevated in SPMS CSF over PPMS CSF. Downstream molecules of EMMPRIN include the MMPs. Serum and CSF MMPs including MMP-9 [23] and MMP-2 [24] strongly associate with disease activity in MS and are known to damage BBB integrity [7]. Also, tumor necrosis factor (TNF)-alpha converting enzyme (TACE, also called ADAM17), another metalloproteinase, was found to be elevated in SPMS monocytes when compared to PPMS monocytes [25]. Indeed, the SPMS brain has relatively compromised BBB compared to the PPMS brain [26]. Therefore, as a potential inducer of MMPs, it is likely that elevated sEMMPRIN levels in SPMS CSF reflect enhanced MMP activities, thus leading to a greater BBB disruption in the SPMS compared to PPMS brain.

The presence of high sEMMPRIN levels in the CSFs of the discectomy group and MS subjects suggest that EMMPRIN is a molecule of high turnover in the CNS and may hold importance to CNS homeostasis. Thus, variations in CSF sEMMPRIN levels in different MS subtypes may indicate homeostatic alterations. Metabolic studies in the MS brain have revealed that lactate (a monocarboxylate) is elevated in the CSF of SPMS subjects compared to normal control subjects [27]. Further, increase in serum lactate also correlates with disease progression in MS [28]. Lactate is an important source of energy for neurons [29], and is transported across cells via the monocarboxylate transporters (MCTs), MCT-1, MCT-2 and MCT-4 [30, 31]. Notably, EMMPRIN is an important chaperone for MCT-1 and MCT-4 [32] and thus, a key player for the regulation of metabolism. Elevated sEMMPRIN levels in the CSF may thus reflect important EMMPRIN functions in the CNS that have yet to be fully elucidated. The apparent difference in sEMMPRIN between SPMS and PPMS CSFs may reflect altered sEMMPRIN functions that require additional characterization in the future. However, as sEMMPRIN levels in the CSF of SPMS subjects are not significantly different from that of controls, the finding that sEMMPRIN is higher in SPMS CSF over PPMS needs to be tempered. Alternately, the CSFs obtained from discectomy controls are not representative of truly normal subjects, so the apparent difference between SPMS and PPMS samples may reflect a pathophysiological process that warrants further attention. Further, as EMMPRIN can be secreted either as a full-length form or as a cleaved fragment, it is important to know if different forms of sEMMPRIN are present in the CSF. However, as the ELISA antibody was generated against the full-length form of EMMPRIN (according to the manufacturer), it is difficult to know whether the ELISA identified the cleaved fragments in addition to recognizing the full-length form of EMMPRIN. Identification of specific sEMMPRIN fragments in body fluids will potentially provide valuable information about the mechanisms of EMMPRIN’s involvement in disease pathology. Also, EMMPRIN can be secreted via extracellular vesicles called exosomes in various pathological conditions including cancer [33, 34]. Since exosomes have been reported to be present in the CSFs of patients with Alzheimer’s disease [35] and Parkinson’s disease [36], it will be informative to study exosomal EMMPRIN in the CSFs of MS subjects.

## Conclusions

To the best of our knowledge, this is the first study highlighting the presence of sEMMPRIN in the CSF. Although EMMPRIN’s role in multiple inflammatory processes has been recognized [37, 38], its soluble form does not appear to be a reliable measure of inflammatory disease activity, particularly in the circulation. CSF level of sEMMPRIN is higher generally than that found in serum, and there appears to be an elevation of sEMMPRIN in CSF in secondary versus primary progressive MS, although these levels were not different from the discectomy group. Despite the lack of promise of sEMMPRIN as a marker of disease activity, the high levels of sEMMPRIN in the CSF suggest important roles of EMMPRIN in the blood-brain interface and within the CNS parenchyma, and this deserves to be investigated further.

## Supporting Information

S1 Fig Stability of CSF sEMMPRIN: Measurement of sEMMPRIN in aliquots of CSFs from five subjects performed at 2 intervals spaced 20 months apart shows that values of sEMMPRIN were similar at the 2 periods of measurements. These samples were stored at -80°C until analyzed. Error bars indicate differences from duplicate wells for each individual ELISA.
(TIF)

S1 Table Main data set of amounts of sEMMPRIN contributing to the findings of this paper. (XLSX)
