# Supplementary figures and images for: Evaluating Soluble EMMPRIN as a Marker of Disease Activity in Multiple Sclerosis: Studies of Serum and Cerebrospinal Fluid
Source: PLoS One. 2016 Oct 11;11(10):e0163802. doi: 10.1371/journal.pone.0163802 (PMC5058493; doi:10.1371/journal.pone.0163802)

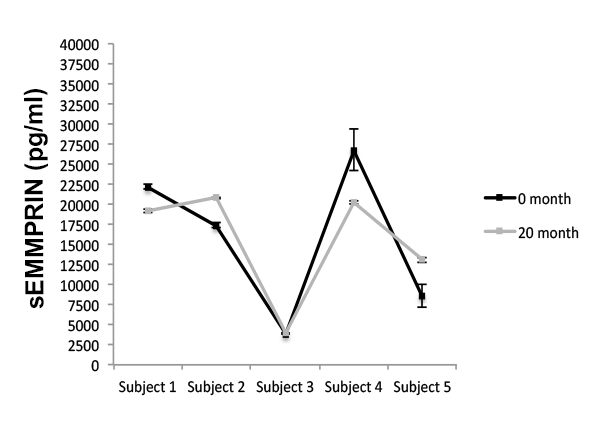

Supplement: S1 Fig — These samples were stored at -80°C until analyzed. Error bars indicate differences from duplicate wells for each individual ELISA. (TIF) [file pone.0163802.s001.tif]
